# Supplementary material for: Effects of Diet on the Gut Bacterial Community of Aldrichina grahami (Diptera: Calliphoridae) across Developmental Stages
Source: Insects. 2024 Mar 7;15(3):181. doi: 10.3390/insects15030181 (PMC10970864; doi:10.3390/insects15030181)
Supplement: Supplementary file 1 [file insects-15-00181-s001.zip › insects-2852905-supplementary.pdf]

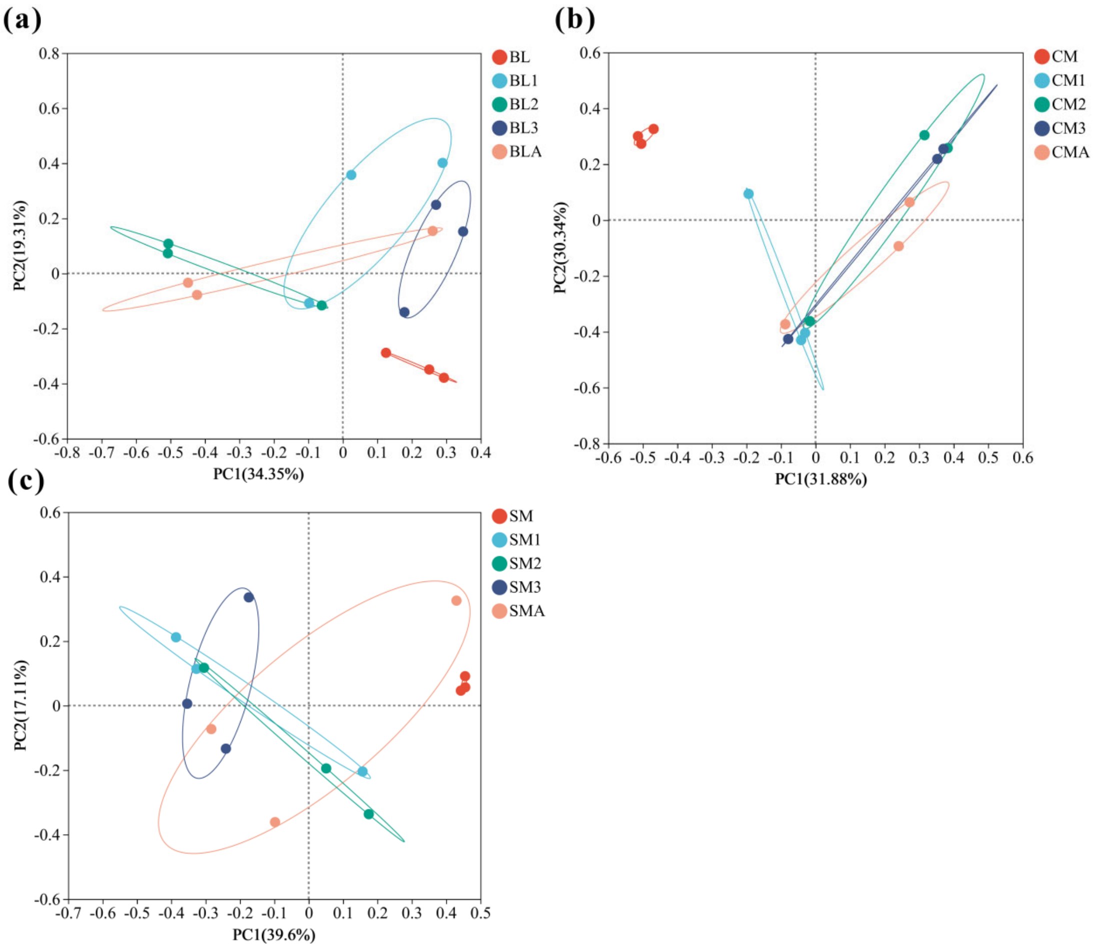

Figure S1 Principal coordinates analysis (PCoA) based on Bray-Curtis distance. (a) diet bovine liver and gut samples obtained from bovine liver group; (b) chicken swine and gut samples obtained from chicken manure group; (c) swine manure and gut samples obtained from swine manure group. BL: bovine liver; CM: chicken manure; SM: swine manure; BL(1-3): 1-3 instar larvae feed by bovine liver; CM (1-3): 1-3 instar larvae feed by chicken manure; SM (1-3): 1-3 instar larvae feed by swine manure diets; BLA, CMA and SMA: newly emerged adults developed on bovine liver, chicken manure and swine manure diets.

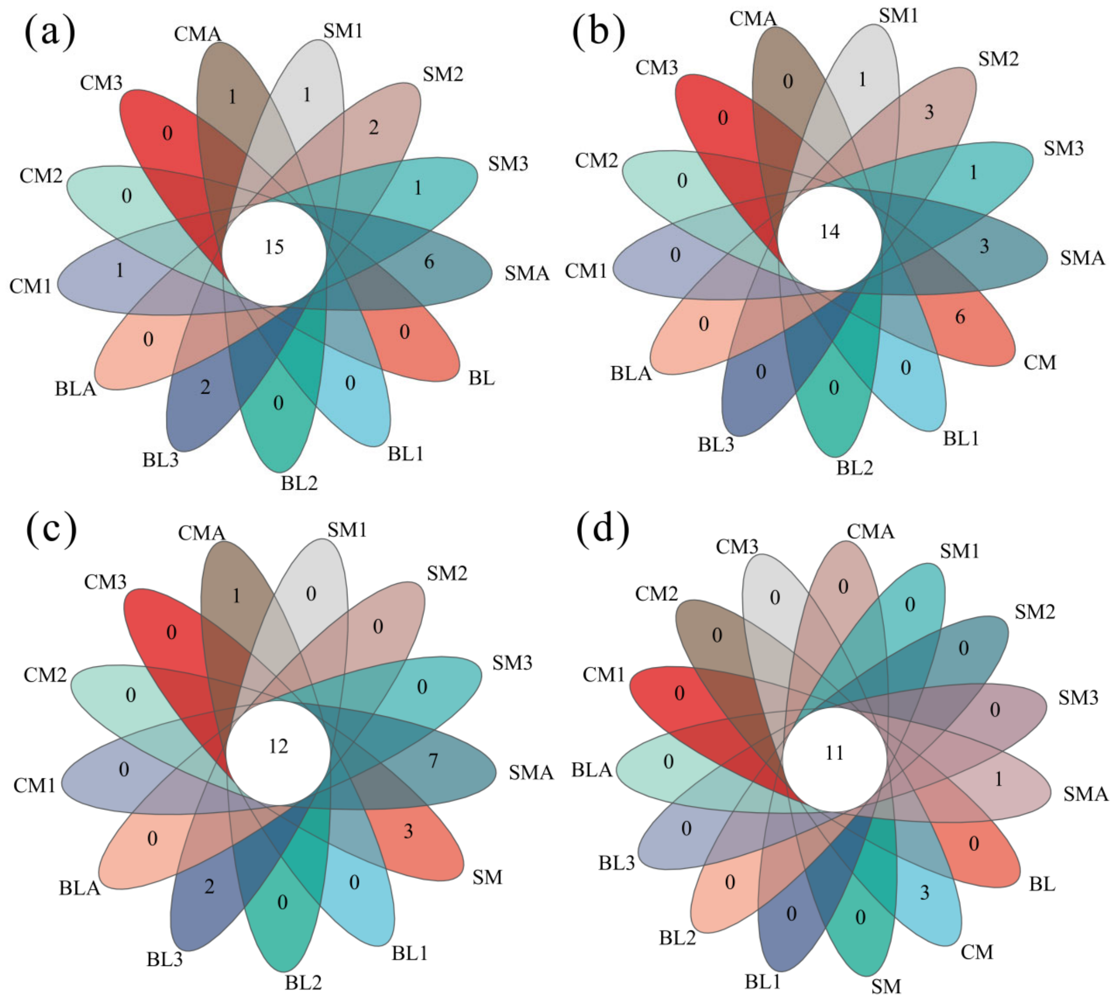

Figure S2 Venn diagrams of microbiome of diets and gut microbiome of *A. grahami* in different treatments. (a) bovine liver and gut samples; (b) chicken liver and gut samples; (c) swine manure and gut samples; (d) three diets and gut samples. BL: bovine liver; CM: chicken manure; SM: swine manure; BL(1-3): 1-3 instar larvae feed by bovine liver; CM (1-3): 1-3 instar larvae feed by chicken manure; SM (1-3): 1-3 instar larvae feed by swine manure diets; BLA, CMA and SMA: newly emerged adults developed on bovine liver, chicken manure and swine manure diets.

Table S1. Sample sequencing data statistics

Diets	Stages	Samples	Optimized sequence	Effective sequence	Average Length	Effective sequence ratio（%）
BL food	-	1	90,177	81,199	429	90
		2	67,030	63,912	428	95
		3	82,096	76,915	429	94
CM food	-	1	49,107	28,838	423	59
		2	72,680	38,877	427	54
		3	43,090	24,298	425	56
SM food	-	1	56,430	36,140	415	64
		2	45,840	28,430	417	62
		3	71,159	42,876	419	60
BL	1st	1	38,963	33,710	427	87
		2	36,568	34,557	429	95
		3	46,938	42,313	427	90
	2nd	1	38,821	36,801	429	95
		2	41,441	39,164	429	95
		3	47,794	46,537	428	97
	3rd	1	36,913	35,304	429	96
		2	41,366	33,924	429	82
		3	53,058	44,952	424	85
	adult	1	42,995	42,518	428	99
		2	38,014	35,897	429	94
		3	50,835	48,150	429	95
CM	1st	1	50,899	50,241	428	99
		2	37,346	33,273	428	89
		3	35,340	33,210	428	94
	2nd	1	39,380	35,571	428	90
		2	55,000	44,914	418	82
		3	50,259	36,651	412	73
	3rd	1	49,220	48,295	429	98
		2	52,331	43,050	419	82
		3	48,704	36,541	417	75
	adult	1	43,731	33,598	429	77
		2	51,458	42,359	420	82
		3	45,491	34,600	422	76
SM	1st	1	46,606	39,035	425	84
		2	40,944	33,118	426	81
		3	34,332	33,572	424	98
	2nd	1	52,434	49,113	429	94
		2	43,112	39,518	420	92
		3	40,569	37,623	423	93

	3rd	1	42,314	33,956	428	80
		2	34,954	34,177	426	98
		3	51,139	44,424	427	87
	adult	1	41,636	33,587	429	81
		2	55,536	54,559	418	98
		3	54,741	44,100	422	81

Table S2. The value of Good’s coverage index in all the samples

Diets	Stages	Samples	Coverage (%)
BL food	-	1	99.93
		2	99.93
		3	99.91
CM food	-	1	99.93
		2	99.92
		3	99.96
SM food	-	1	99.93
		2	99.92
		3	99.92
BL	1st	1	99.94
		2	99.96
		3	99.97
	2nd	1	99.97
		2	99.98
		3	99.99
	3rd	1	99.97
		2	99.97
		3	99.94
	adult	1	99.99
		2	99.98
		3	99.99
CM	1st	1	99.99
		2	99.96
		3	99.95
	2nd	1	99.95
		2	99.95
		3	99.95
	3rd	1	99.97
		2	99.97
		3	99.95
	adult	1	99.97
		2	99.95
		3	99.95
SM	1st	1	99.95
		2	99.95
		3	99.95
	2nd	1	99.99
		2	99.96
		3	99.95
	3rd	1	99.95

		2	99.96
		3	99.97
	adult	1	99.97
		2	99.98
		3	99.95

Table S3. Coexistence of bacterial genera depicted in the Venn diagram

No.	Gut samples	Bovine liver	Chicken manure	Swine manure	All the three diets
1	Acinetobacter	+	+	+	+
2	Carnobacterium	+	+	+	+
3	Corynebacterium_1	+	+	+	+
4	Enterococcus	+	+	+	+
5	Escherichia-Shigella	+	+	+	+
6	Lactobacillus	+	+	+	+
7	Lactococcus	+	+	+	+
8	Leuconostoc	+	+	+	+
9	Morganella	+	-	-	-
10	Myroides	+	+	-	-
11	Providencia	+	+	-	-
12	Sphingobacterium	+	+	-	-
13	Stenotrophomonas	-	-	+	-
14	unclassified_Enterobacteriaceae	+	+	+	+
15	unclassified_Enterococcaceae	+	+	+	+
16	Vagococcus	+	+	+	+

Note: + means coexistence with gut samples, - means non-coexistence with gut samples.

Table S4. The results of Mann–Whitney U post hoc test for the relative abundance of the two major phyla and the four genera across diets and generations

	Food	1st stage	2ed stage	3rd stage	adult
Firmicutes					
BL	77.37±9.13	53.53±9.31	23.86±8.28	64.60±9.60	40.31±28.09
CM	67.94±3.70	18.35±8.34	69.05±17.39	69.07±22.53	68.70±12.43
SM	81.53±5.55	56.12±11.32	89.84±2.21	68.83±22.72	71.47±4.87
Proteobacteria					
BL	16.59±3.68	36.82±8.78	73.83±9.28	31.51±10.14	59.09±28.65
CM	20.19±7.53	67.61±1.83	21.51±15.97	28.00±22.83	30.23±12.89
SM	11.14±5.55	35.45±9.80	7.33±1.56	25.36±17.51	18.85±2.66
Bacteroidetes					
BL	5.67±5.54	9.40±2.68	2.11±0.90	2.37±1.20	0.03±0.02
CM	0.04±0.02	12.67±8.12	8.10±3.98	2.54±1.49	0.89±0.40
SM	0.02±0.01	6.76±2.24	1.92±1.12	5.15±4.70	1.30±1.29
Actinobacteria					
BL	0.37±0.17	0.23±0.07	0.19±0.11	1.46±+1.22	0.56±0.56
CM	11.39±3.69	1.36±0.65	1.30±0.60	0.36±0.14	0.18±0.06
SM	7.26±0.53	1.61±0.67	0.90±0.47	0.20±0.20	8.22±8.08
Vagococcus					
BL	1.93±0.86	39.46±13.00	16.73±6.33	29.45±12.47	4.44±4.43
CM	2.01±0.63	2.85±1.42	16.35±10.15	32.69±5.98	11.89±4.26
SM	8.94±0.59	30.14±2.37	38.62±2.45	29.36±12.07	19.53±16.41
Providencia					
BL	9.53±2.30	11.9±2.03	15.13±11.71	10.15±1.57	17.28±8.64
CM	0.004±0.002	32.39±16.45	8.45±8.11	19.68±19.36	15.94±7.87
SM	0	24.47±9.02	1.26±0.15	12.95±6.46	1.95±1.72
Morganella					
BL	0.49±0.26	13.83±7.99	53.9±20.58	2.77±1.70	41.07±20.70
CM	0	3.50±1.76	4.04±1.74	2.91±1.40	4.93±2.91
SM	0	3.20±0.16	1.03±0.57	3.32±3.25	1.27±0.79
Lactobacillus					
BL	15.19±6.67	5.15±4.63	3.84±3.84	23.89±10.40	1.56±1.56
CM	15.78±4.46	9.91±5.10	10.52±5.56	7.31±5.41	24.2±6.44
SM	12.28±0.41	9.06±4.13	14.78±3.49	31.62±19.61	17.1±8.53
